# Aminopyridines Restore Ventilation and Reverse Respiratory Acidosis at Late Stages of Botulism in Mice[Fn fn4]

**DOI:** 10.1124/jpet.123.001773

**Published:** 2024-02

**Authors:** William T. McClintic, Zachary D. Chandler, Lalitha M. Karchalla, Celinia A. Ondeck, Sean W. O’Brien, Charity J. Campbell, Alan R. Jacobson, Patrick M. McNutt

**Affiliations:** Wake Forest Institute for Regenerative Medicine, Wake Forest University Health Sciences, Winston-Salem, North Carolina

## Abstract

Botulinum neurotoxin (BoNT) is a potent protein toxin that causes muscle paralysis and death by asphyxiation. Treatments for symptomatic botulism are intubation and supportive care until respiratory function recovers. Aminopyridines have recently emerged as potential treatments for botulism. The clinically approved drug 3,4-diaminopyridine (3,4-DAP) rapidly reverses toxic signs of botulism and has antidotal effects when continuously administered in rodent models of lethal botulism. Although the therapeutic effects of 3,4-DAP likely result from the reversal of diaphragm paralysis, the corresponding effects on respiratory physiology are not understood. Here, we combined unrestrained whole-body plethysmography (UWBP) with arterial blood gas measurements to study the effects of 3,4-DAP, and other aminopyridines, on ventilation and respiration at terminal stages of botulism in mice. Treatment with clinically relevant doses of 3,4-DAP restored ventilation in a dose-dependent manner, producing significant improvements in ventilatory parameters within 10 minutes. Concomitant with improved ventilation, 3,4-DAP treatment reversed botulism-induced respiratory acidosis, restoring blood levels of CO_2_, pH, and lactate to normal physiologic levels. Having established that 3,4-DAP–mediated improvements in ventilation were directly correlated with improved respiration, we used UWBP to quantitatively evaluate nine additional aminopyridines in BoNT/A-intoxicated mice. Multiple aminopyridines were identified with comparable or enhanced therapeutic efficacies compared with 3,4-DAP, including aminopyridines that selectively improved tidal volume versus respiratory rate and vice versa. In addition to contributing to a growing body of evidence supporting the use of aminopyridines to treat clinical botulism, these data lay the groundwork for the development of aminopyridine derivatives with improved pharmacological properties.

**SIGNIFICANCE STATEMENT:**

There is a critical need for fast-acting treatments to reverse respiratory paralysis in patients with botulism. This study used unrestrained, whole-body plethysmography and arterial blood gas analysis to show that aminopyridines rapidly restore ventilation and respiration and reverse respiratory acidosis when administered to mice at terminal stages of botulism. In addition to supporting the use of aminopyridines as first-line treatments for botulism symptoms, these data are expected to contribute to the development of new aminopyridine derivatives with improved pharmacological properties.

## Introduction

Botulinum neurotoxins (BoNT), which cause the neuroparalytic disease of botulism, are a family of protein poisons produced by the *Clostridium* genus of anaerobic bacteria (Pirazzini et al., 2017). BoNTs are the most potent biologic toxins known, with estimated human lethal doses as low as 1–10 ng/kg. Although botulism is relatively rare in humans, with approximately 150 cases per year in the United States (https://www.cdc.gov/botulism/surveillance.html), large-scale intoxication can result from accidental or deliberate exposure to contaminated foods or aerosols (Wein and Liu, 2005; Villar et al., 2006). BoNTs are classified by the Centers for Disease Control and Prevention as tier 1 select agents with a serious risk of use as bioweapons due to their high potential to cause large-scale disruptions and mass casualties (Centers for Disease Control and Prevention and Department of Health and Human Services, 2017). However, despite the urgent need for medical countermeasures, there are no specific treatments for clinical botulism.

Botulism can result from the ingestion of contaminated foods, inhalation or injection of BoNT, or bacterial production of BoNT in wounds or the intestine (Pirazzini et al., 2017). In all cases, systemic disease is caused by the distribution of BoNT in the bloodstream. After selective binding of BoNT to receptors on the presynaptic membrane of peripheral autonomic and motor neurons, the toxin catalytic domain enters the nerve terminal cytosol, where it specifically cleaves presynaptic soluble *N*-ethylmaleimide–sensitive factor attachment protein receptor (SNARE) proteins essential for synaptic vesicle fusion and neurotransmitter release (Burgen et al., 1949; Simpson, 2004; Montal, 2009). As the number of cleaved SNARE proteins increases, neurotransmission deteriorates, resulting in progressive muscle weakness that culminates in flaccid paralysis. At lethal doses, botulism typically presents with cranial nerve palsies after a 12–36-hour latent period, followed by a bilateral descending paralysis that progresses to respiratory arrest by 3 days (Lindström and Korkeala, 2006; Witoonpanich et al., 2010). The rate of disease progression and extent of neuromuscular paralysis are proportional to the BoNT dose. Survival can be maintained through sustained artificial ventilation and parenteral nutrition until respiratory paralysis resolves, which can take weeks to months. Death is typically the result of early respiratory arrest or complications from prolonged intensive care, such as ventilator-associated pneumonia and deep vein thrombosis (Sobel, 2005).

The only approved pharmacotherapy for botulism is post-exposure prophylaxis with antibotulinum antibodies (a.k.a. antitoxin), which terminate exposure by neutralizing the toxin in the bloodstream. However, respiratory motor neurons often internalize paralytic amounts of BoNT during the latent period between exposure and symptomatic manifestation. Consequently, approximately 70% of patients who receive antitoxin after symptomatic emergence ultimately require mechanical ventilation for survival (Yu et al., 2017; Richardson et al., 2020). The development of small-molecule inhibitors that block toxin activity within the neuron has proven challenging due to various factors, including the large interface that forms between the toxin and its proteolytic target, the extensive conformational flexibility of the toxin catalytic domain, and the need for multiple small molecules to target diverse toxin serotypes (Breidenbach and Brunger, 2004; Silvaggi et al., 2007; Kumar et al., 2014). Recent reports demonstrate that intraneuronal delivery of therapeutic antibodies can have antidotal effects in animal models of botulism (McNutt et al., 2021; Miyashita et al., 2021); however, these treatments are still in preclinical development. It should be noted that antidotes that directly inhibit molecular toxicity will have delayed benefits on botulism symptoms because neuromuscular recovery will only occur once sufficient SNARE proteins are regenerated to enable reliable neurotransmission, which may take days (Bartels et al., 1994). Consequently, there is an urgent need for a fast-acting symptomatic treatment that 1) maintains respiratory function until the patient can receive definitive care and 2) reduces reliance on mechanical ventilation.

3,4-Diaminopyridine (3,4-DAP) is a clinically approved treatment of Lambert-Eaton myasthenic syndrome (LEMS), which is an autoimmune disease caused by reduced acetylcholine release and neuromuscular weakness (McEvoy et al., 1989). We recently demonstrated that 3,4-DAP is also a potent reversal agent for respiratory paralysis caused by botulism. 3,4-DAP prolongs action potential duration by reversibly blocking voltage-gated potassium channels (VGKC), facilitating presynaptic Ca^2+^ influx and increasing acetylcholine release (Kirsch and Narahashi, 1978; Thomsen and Wilson, 1983; Ojala et al., 2021). Mechanistic studies revealed that 3,4-DAP restores neurotransmission at intoxicated neuromuscular junctions (NMJs) by increasing the number of sites activated to release acetylcholine during an action potential (Bradford et al., 2018). Therefore, by increasing the release of acetylcholine, 3,4-DAP directly addresses the pathophysiology caused by botulism. Indeed, 3,4-DAP restores phrenic neurotransmission and nerve-evoked muscle contractions in isolated mouse diaphragms poisoned by multiple BoNT serotypes, with improved efficacy for BoNT/A (Bradford et al., 2018). Administration of human-equivalent doses of 3,4-DAP rapidly reverses toxic signs and prolongs survival in mice at terminal stages of botulism (Vazquez-Cintron et al., 2020), whereas continuous infusion of 3,4-DAP has antidotal efficacy in rats challenged with lethal doses of BoNT/A (Machamer et al., 2022).

Although 3,4-DAP is presumed to treat botulism symptoms by improving respiratory function, the specific impact of 3,4-DAP on ventilation and respiratory physiology remains unknown. Here, we combined unrestrained whole-body plethysmography (UWBP) with arterial blood gas measurements to quantitatively assess the dose-dependent effects of 3,4-DAP, as well as nine additional aminopyridines, on respiratory function in mice at terminal stages of botulism. Mice were challenged with BoNT serotype A, which is responsible for approximately half of natural botulism cases in the United States (Sobel et al., 2004). BoNT/A is also the active component in most BoNT-based pharmaceuticals (Foran et al., 2003; Brashear, 2008; Pirazzini et al., 2017), thus positioning aminopyridines as potential treatments for botulism symptoms caused by natural or iatrogenic exposures. Because neurons intoxicated by BoNT/A are more sensitive to the therapeutic effects of 3,4-DAP than neurons intoxicated by other serotypes (Beske et al., 2016, 2017; Bradford et al., 2018), we limited this study to the use of BoNT/A to increase the chances of identifying pharmacologic effects.

Our results highlight the therapeutic effects of clinically relevant doses of 3,4-DAP on respiratory acidosis in intoxicated mice. Furthermore, we identify other aminopyridines with comparable or enhanced therapeutic effects compared to 3,4-DAP. The identification of new aminopyridines that are therapeutically effective in a preclinical model of lethal botulism illustrates the power of this approach for drug discovery in botulism and other neuromuscular and respiratory diseases. These findings provide further information on the potential of aminopyridines as primary treatments for botulism symptoms and inform the development of new aminopyridine derivatives with improved pharmacological properties for the treatment of botulism as well as other myasthenias caused by impaired neurotransmission.

## Materials and Methods

### Animals

Female CD-1 mice (*n* = 248, 8–12 weeks of age; Charles River Laboratories) were group housed, maintained on a 12-hour diurnal cycle, and provided a standard diet with regular enrichment and water ad libitum. We have not observed any difference in intoxication or treatment with 3,4-DAP between male and female mice. Female mice were used for this study to provide historical continuity and capture the inherent variability present throughout estrous. For intoxication, mice weighing 23–29 g were randomly assigned to groups. Mice were euthanized with 3% isoflurane followed by exsanguination or cervical dislocation. All studies are reported consistent with the ARRIVE 2.0 guidelines (Percie du Sert et al., 2020), and all animal use procedures were conducted in accordance with the principles stated in the Guide for the Care and Use of Laboratory Animals and the Animal Welfare Act of 1966 (Public Law 89-544, as amended in Public Law 115-334). The experimental protocol was approved by the Animal Care and Use Committee at the Wake Forest University School of Medicine (United States Department of Agriculture certificate number 55-B-0182).

### Reagents

Botulinum neurotoxin serotype A1 (2.5 × 10^8^ LD_50_/mg) was purchased from Metabiologics, Inc. (Madison, WI) at 10 µg/mL and stored at 4°C. BoNT/A was diluted to working concentrations in PBS with 0.2% gelatin (ThermoFisher, Waltham, MA) and administered by tail vein injection using a 27-gauge needle. The potency of each toxin lot was measured using the mouse lethality assay described previously (Vazquez-Cintron et al., 2020). Survival rates at 96 hours after intoxication were used to calculate LD_50_ values using simple logistic regression. Intravenous LD_50_ values for the two lots of BoNT/A used in this study were determined to be 0.175 pg/g (95% CI: 0.154–0.196) and 0.215 pg/g (95% CI: 0.191–0.239). The aminopyridines used in this study include 4-aminopyridine (4-AP; Sigma Aldrich, St. Louis, MO), 3,4-DAP (Fisher Scientific, Hampton, NH), 2,4-diaminopyridine (2,4-DAP; Santa Cruz Biotechnology, Inc., Dallas, TX), 3,4,5-triaminopyridine (3,4,5-TAP; Ambeed, Arlington Heights, IL), deuterated 4-aminopyridine-d4 (4-AP-d4; Millipore Sigma), deuterated 3,4-diaminopyridine-d3 (3,4-DAP-d3; Toronto Research Chemicals, Ontario, CA), 4-amino-3-fluoropyridine (3F-4AP; Millipore Sigma), 4-amino-2-fluoropyridine (2F-4AP; Ambeed), 3-methyl-4-aminopyridine (3Me-4AP, Ambeed), and 4-amino-3,5-difluoropyridine (3,5DF-4AP; Ambeed). Aminopyridines were diluted to working concentrations with PBS (pH 7.4; Sigma Aldrich) prior to injection.

### Plethysmography

Ventilation parameters were monitored using unrestrained whole-body plethysmography in a four-chamber vivoFlow system (Scireq, Montreal Canada). To increase validity, each in vivo study included an intoxicated and vehicle-treated cohort. Mice were habituated to the plethysmography chambers at least three times at 24-hour intervals before intoxication with BoNT. Mice were intoxicated as above and monitored for progression of symptoms. The next day, mice that met the toxic signs criteria (severe abdominal paradox and extreme lethargy) were randomly assigned to treatment groups and baseline assessments of ventilation parameters were made. Mice were entered into the experimental protocol once they exhibited the above toxic signs. The average minute volume (MV) of mice used in plethysmography (*n* = 186) and blood gas studies (*n* = 62) was 10.84 ± 5.7 mL/min (mean ± S.D.), and 50% of mice had minute volumes between 6.3–14.4 mL/min (Supplemental Table 1). After baseline recordings, mice were administered vehicle (Dulbecco’s phosphate buffered saline) or an aminopyridine via a 200-uL subcutaneous injection above the scapula using a 25-gauge needle and returned to the plethysmography chamber for continuous monitoring for an additional 90 minutes. Chambers were cleaned with REScue veterinary-grade disinfectant (Virox Animal Health, Ontario, Canada) between mouse runs. IOX software (2.10.8.25; emka Technologies S.A.S., Paris, France) was used to sample flow data at 1000 Hz and detect respiratory events. Respiratory events were recorded with a flow threshold of 0.8 mL/s and rejected if the ratio of inspired-to-expired volume exceeded 80%. The raw signal was digitally smoothed with a 20-ms time constant and a high-pass filter of 4000 millisecond to compensate for baseline drift. Ventilatory parameters were recorded over minute intervals and subsequently binned into 5-minute intervals for analysis. After completion of acquisition, a custom Python script was used to extract the ventilation parameter data from the output text file for further analysis in Graphpad Prism version 9.4 (Graphpad Software, San Diego, CA). Flow rates into and out of each chamber were verified weekly using a digital flowmeter and calibrated each day prior to recording.

### Arterial Blood Gas Measurements

For arterial blood gas measurements, mice were anesthetized with 3% isoflurane in 97% room air through a nosecone at 286 mL/min (Somnosuite, Kent Scientific, Torrington, CT). Full sedation was verified by toe pinch. Ethanol was used to sterilize the needle entry site, and 0.2–1 mL of cardiac blood was collected using a 1-mL heparinized syringe with a 25-gauge, 5/8-inch needle and immediately analyzed using a blood gas analyzer (Nova Biomedical, Waltham, MA). Sample collection was consistently between 2.5–3.0 minutes after the start of anesthesia. After sample collection, mice were sacrificed by cervical dislocation.

### Toxicity Studies

Mice (*n* = 3 per aminopyridine) were administered subcutaneous injections with each experimental aminopyridine at 2 mg/kg and scored for signs of toxicity such as gait change, salivation, or behavioral changes for 1.5 hours. To characterize potential central nervous effects, median behavioral signs of seizure were estimated using the seven-point Racine scale (Lüttjohann et al., 2009).

### Statistics and Data Analysis

All data are presented as mean ± S.E.M. unless otherwise noted. Longitudinal changes in plethysmography data were compared using two-way repeated measures ANOVA Geisser-Greenhouse corrections and the Šídák’s or Dunnett’s multiple comparison test as indicated. Blood gas values of arterial carbon dioxide partial pressure (*p*CO_2_), lactate, bicarbonate, pH, and arterial oxygen partial pressure (*p*O_2_) for each condition were compared with vehicle-treated samples using ordinary one-way ANOVA and Dunnett’s multiple comparison test against vehicle-treated data. Binary comparisons of mean *p*O_2_ were made using Welch’s *t* test. Nonlinear regressions between 3,4-DAP dose and peak area-under-the-curve values for tidal volume (TV), minute volume, and respiratory rate (RR) were fit using a four-parameter variable slope curve. An adjusted value of *P* < 0.05 was considered significant in all comparisons. Statistical comparisons were made using Graphpad Prism v9.4. Additional details of statistical tests, significance values, and numbers of samples are included in Supplemental Table 1.

## Results

### 3,4-DAP (s.c., 2 mg/kg) Improves Ventilation and Reverses Respiratory Acidosis at Terminal Stages of Botulism

**Fig. 1.**
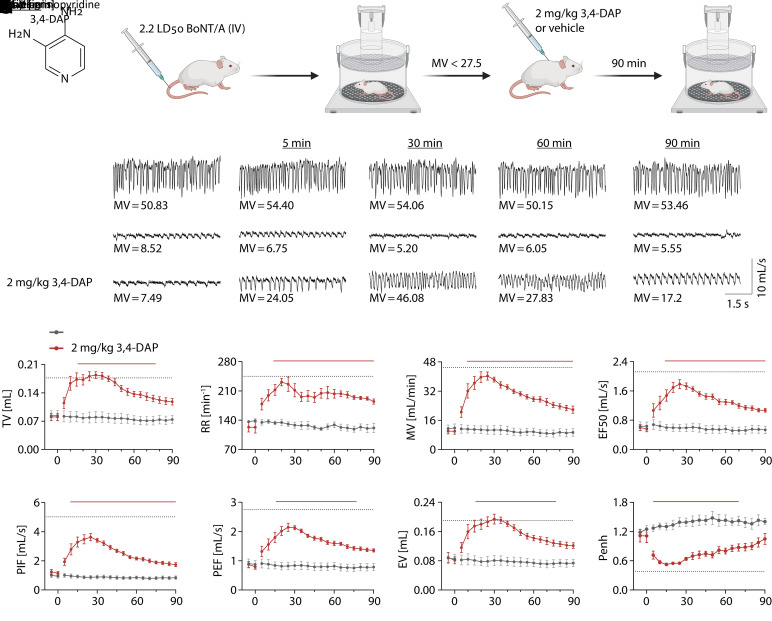
3,4-DAP (2 mg/kg, s.c.) improves ventilatory parameters in mice at terminal stages of botulism. Mice were challenged with 2.2x LD_50_ BoNT/A (i.v.) and observed for development of toxic signs of botulism. Following baseline plethysmography measurements, mice were treated with 2 mg/kg 3,4-DAP (*n* = 6) or vehicle (*n* = 6) by subcutaneous injection. Mice were immediately returned to plethysmography chambers, and ventilation was quantified over 90 minutes. (A) Structure of 3,4-DAP. (B) Cartoon of experimental procedures. (C) Representative 6-second traces collected during baseline and at 5, 30, 60, and 90 minutes after the start of observation from naïve mice (top panels; maintained in plethysmography chambers without treatment), mice that were intoxicated and treated with vehicle (middle panels), and mice that were intoxicated and treated with 3,4-DAP (bottom panels). The corresponding MV is presented below each trace. Equal scaling was used for all traces. (D–K) Mean ± S.E.M. for each ventilatory parameter after vehicle or 3,4-DAP treatment, including TV (D), RR (E), MV (F), mid-expiratory flow (G), peak inspiratory flow (H), peak expiratory flow (I), expired volume (J), and enhanced pause (K). Dotted lines in each graph represent the 95% confidence interval of the mean value for each parameter. Solid lines represent time points that are significantly different than vehicle (*P* < 0.05; repeated measures two-way ANOVA with Sidak’s multiple comparison test). Details of statistical comparisons are provided in Supplemental Table 1. EF50, mid-expiratory flow; EV, expired volume; Penh, enhanced pause; PEF, peak expiratory flow; PIF, peak inspiratory flow.

We previously reported that subcutaneous administration of human-equivalent doses of 3,4-DAP improved respiratory rate, toxic signs, and overall activity in mice in terminal stages of botulism (Vazquez-Cintron et al., 2020). To develop a more rigorous evaluation of the effects of 3,4-DAP on respiratory function, UWBP was used to quantify ventilation in mice challenged with 2.2x LD_50_ BoNT/A (i.v.) and treated with vehicle or 3,4-DAP (Fig. 1, A and B). These initial studies used 2 mg/kg 3,4-DAP to allow direct comparison with previous reports showing improved toxic signs and activity in intoxicated mice and rats at the same dose (Vazquez-Cintron et al., 2020; Machamer et al., 2022).

Terminal signs of botulism emerged approximately 18–30 hours after intoxication, manifesting as severe abdominal paradox, lethargy, and muscle weakness. Plethysmography of mice with severe abdominal paradox revealed marked declines in ventilatory parameters compared with naïve mice (Fig. 1C), including reduced MV (10.8 ± 0.7 mL/min versus 48.1 ± 1.5 mL/min; *P* < 0.0001, Welch’s *t* test), TV (0.082 ± 0.002 mL versus 0.188 ± 0.006 mL; *P* < 0.0001, Welch’s *t* test), and RR (129.9 ± 6.6 minute^−1^ versus 260.9 ± 8.0 minute^−1^; *P* < 0.0001, Welch’s *t* test). Although vehicle treatment had no effect on ventilation, 3,4-DAP significantly improved a wide range of ventilatory parameters, including TV, RR, MV, mid-expiratory flow, peak inspiratory flow, peak expiratory flow, expired volume, and enhanced pause between breaths (Fig. 1, D–K). MV, which is the product of TV and RR, reflects total gas exchange per minute and thus is the most comprehensive summary of ventilatory function. MV significantly improved 10 minutes after 3,4-DAP treatment and reached maximum effect 25–30 minutes after 3,4-DAP treatment (Fig. 1F). Consistent with previous findings, the clinical benefits of a single administration of 3,4-DAP were transient, and ventilatory parameters began to decline after approximately 30 minutes. However, compared with vehicle treatment, all ventilatory parameters remained significantly improved up to 90 minutes after treatment.

**Fig. 2.**
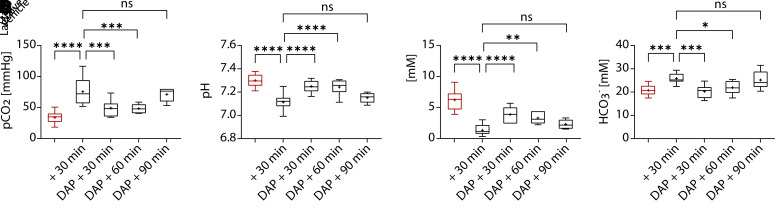
3,4-DAP (2 mg/kg, s.c.) improves blood gas levels in mice at terminal stages of botulism. Mice were challenged with 2.2x LD_50_ BoNT/A (i.v.) and observed for toxic signs of botulism. Following baseline recordings to establish minute volumes, intoxicated mice were treated s.c. with vehicle (*n* = 17) or 2 mg/kg 3,4-DAP (*n* = 7 at 30 minutes, *n* = 6 at 60 minutes, *n* = 6 at 90 minute). For comparison, cardiac blood was also analyzed from naïve mice (*n* = 12). At the indicated times after treatment, mice were sedated, and cardiac samples were analyzed for (A) *p*CO_2_, (B) pH, (C) lactate, and (D) HCO_3_^−^. Details of statistical comparisons are provided in Supplemental Table 1. *P < 0.05; **P < 0.01; ***P < 0.001; ****P < 0.0001. ns, not significant.

Respiratory arrest is the primary mode of death in patients with botulism who do not receive intensive care support (Sobel, 2005; Rao et al., 2021). To determine whether treatment with 3,4-DAP also affected respiration, we measured blood gas levels in cardiac samples collected from naïve mice or intoxicated mice treated with vehicle or 2 mg/kg of 3,4-DAP. Naïve blood gas values were consistent with previous reports (Fig. 2, A–D) (Iversen et al., 2012). Vehicle-treated mice exhibited characteristic markers of hypercapnic-induced respiratory acidosis, including significant increases in *p*CO_2_ (Fig. 2A) and bicarbonate (HCO_3_^-^; Fig. 2B), reduced pH (Fig. 2C), and increased lactate (Fig. 2D). Treatment with 3,4-DAP reversed respiratory acidosis within 30 minutes, with significant improvements in *p*CO_2_, HCO_3_^-^, pH, and lactate (Fig. 2, A–D). To assess whether 3,4-DAP treatment improved respiration for the same duration as ventilation, we measured blood gas values at 60 and 90 minutes after treatment (Fig. 2, A–D). Although blood gas levels remained significantly improved versus vehicle at 60 minutes, therapeutic effects were lost by 90 minutes. The reversal of therapeutic effects on blood gas levels at 90 minutes was consistent with previous behavioral studies showing loss of symptomatic benefits between 60 and 90 minutes after single administration of 2 mg/kg 3,4-DAP (Vazquez-Cintron et al., 2020). The loss of physiologic benefits after 60 minutes highlights the need for treatment strategies that sustain maximum increases in ventilation for extended periods.

Neither intoxication nor 3,4-DAP treatment had an effect on *p*O_2_ levels [*F*(6, 55) = 1.69, *P* = 0.14, ordinary one-way ANOVA]. To determine whether *p*O_2_ levels decreased at later stages of botulism, we measured blood gas levels in mice that were moribund with severe abdominal paradox, agonal breathing, and >90% reduction in MV (i.e., <4.4 ± 0.6 mL/min). Despite the severity of respiratory signs, *p*O_2_ was not reduced in moribund mice compared with naïve mice (67.7 ± 15.1 mmHg versus 77.6 ± 8.1 mmHg; *n* = 8 each, *P* = 0.47, Welch’s *t* test). These data suggest that respiratory acidosis is an early manifestation of botulism, whereas hypoxemia develops shortly before death.

### 3,4-DAP Produces Dose-Dependent Clinical Benefits

**Fig. 3.**
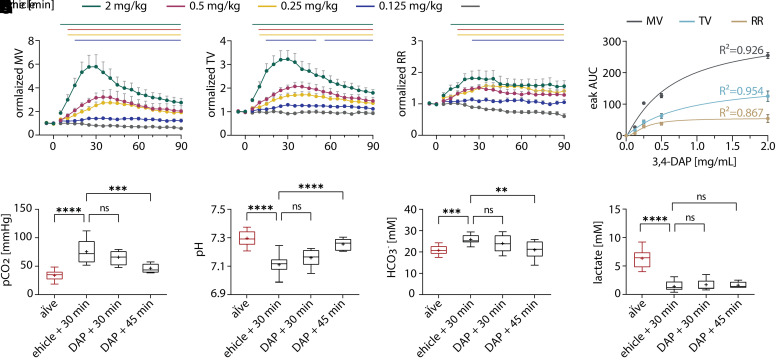
Dose-dependent effects of 3,4-DAP on respiratory parameters and blood gas levels. Mice were challenged with 2.2x LD_50_ BoNT/A (i.v.) and monitored for respiratory signs of botulism. Following baseline recordings, mice were treated s.c. with vehicle (*n* = 6) or 3,4-DAP at 0.125 mg/kg (*n* = 11), 0.25 mg/kg (*n* = 12), 0.50 mg/kg (*n* = 10), or 2.0 mg/kg (*n* = 17). (A–C) Ventilatory parameters were measured for 90 minutes after treatment, normalized to baseline values for each mouse, and reported as mean MV (A), TV (B), and RR (C). Solid lines represent time points that are significantly different than vehicle (*P* < 0.05; repeated measures two-way ANOVA with Dunnett’s multiple comparison test against vehicle). (D) Mean area-under-the-curve values for MV, TV, and RR peaks were plotted against 3,4-DAP dose and fit with a nonlinear regression. The resulting goodness-of-fit values (R^2^) are presented above each fit. (E–H) Blood gas measurements of *p*CO_2_ (E), pH (F), HCO_3_^−^ (G), and lactate (H) were made at 30 and 45 minutes after treatment. Details of statistical comparisons are provided in Supplemental Table 1. **P < 0.01; ***P < 0.001; ****P < 0.0001. ns, not significant.

In mice, 2 mg/kg 3,4-DAP is the allometric equivalent of the Food and Drug Administration–approved 10 mg clinical dose of 3,4-DAP (amifampridine phosphate). However, subcutaneous administration of 2 mg/kg 3,4-DAP in mice results in maximum blood levels of 840 ng/mL (Vazquez-Cintron et al., 2020), which is markedly higher than maximum blood levels in humans after oral administration of 10 mg amifampridine (38.6 ± 9.2 ng/mL) (Haroldsen et al., 2017). To determine the lowest dose of 3,4-DAP that improves respiratory function, we measured the dose-dependent effects of 3,4-DAP treatment on ventilatory parameters in intoxicated mice. Mice with respiratory signs of botulism were treated with vehicle or 3,4-DAP at 0.125, 0.25, 0.5, or 2.0 mg/kg, and ventilation was measured as described above (Fig. 1B). 3,4-DAP evoked dose-dependent improvements in ventilation (Fig. 3, A–C). Although MV, TV, and RR show dose-dependent responses to 3,4-DAP, MV was the most sensitive ventilatory parameter (Fig. 3D). To assess whether lower doses of 3,4-DAP still produced therapeutic effects on respiration, blood gas levels were measured after treatment with 0.25 mg/kg 3,4-DAP, which was the lowest dose to produce an unambiguous peak response in MV. Although blood gas levels did not improve by 30 minutes after treatment with 0.25 mg/kg 3,4-DAP, *p*CO_2_, pH, and HCO_3_^-^ improved by 45 minutes after treatment, coinciding with the maximum improvement in MV (Fig. 3, E–H). Lactate did not show any significant improvement at this dose even after 45 minutes post-treatment. These data confirmed that doses ≥0.25 mg/kg 3,4-DAP increased both ventilatory parameters and respiration in mice with severe botulism.

### Evaluation of 4-Aminopyridine Derivatives on Ventilation in Intoxicated Mice

Clinical dosing of 3,4-DAP is complicated by a short half-life, a sixfold difference in exposure caused by phenotypic differences among patients, and a relatively narrow therapeutic index. Consequently, 3,4-DAP is typically administered to LEMS patients from 3 to 6 times a day at relatively low doses to avoid adverse effects (Thakkar et al., 2017). Previous studies conducted in animal models of botulism lead us to hypothesize that reversal of botulism symptoms requires approximately three- to fourfold higher exposures than are needed for typical patients with LEMS (125 ng/mL versus 32 ng/mL 3,4-DAP) (Thakkar et al., 2017; Vazquez-Cintron et al., 2020; Machamer et al., 2022). Consequently, there is significant interest in identifying small molecules with more suitable physicochemical properties for the treatment of botulism and other neuromuscular indications. However, few attempts have been made to evaluate other aminopyridines as symptomatic treatments for botulism (Ball et al., 1979; Mayorov et al., 2010).

**Fig. 4.**
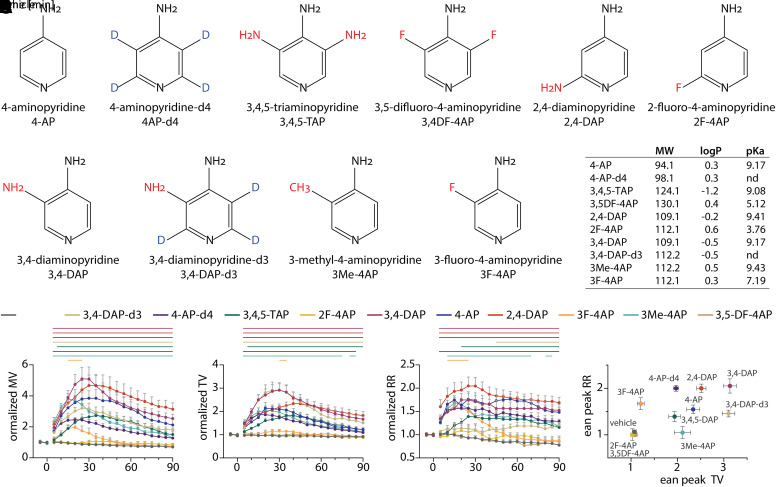
Evaluation of aminopyridine derivatives on ventilation. Mice were challenged with 2.2x LD_50_ BoNT/A (i.v.) and monitored for respiratory signs of botulism. Once mice developed severe abdominal paradox, baseline measurements were made, and mice were treated s.c. with vehicle (*n* = 14), 3,4-DAP (*n* = 25), 2,4-DAP (*n* = 16), 3,4,5-TAP (*n* = 12), 4-AP (*n* = 11), deuterated 4-aminopyridine (4-AP-d4) (*n* = 11), 3,4-DAP-d3 (*n* = 8), 3F-4AP (*n* = 8), 2-fluoro-4-aminopyridine (2F-4AP) (*n* = 6), or 3,4DF-4AP (*n* = 3) and monitored for 90 minutes. (A) Structures of experimental aminopyridines. 3,4-DAP is included for comparison. (B) Summary of physicochemical properties. Lipophilicity (LogP) values were derived from Pubchem (https://pubchem.ncbi.nlm.nih.gov), whereas negative logarithm of the acid dissociation constant (pK_a_) values were derived from Chemicalbook (https://www.chemicalbook.com). All values were confirmed against published literature when available. (C–E). Ventilatory recordings were normalized to baseline values for each mouse, averaged within each treatment condition, and presented as normalized MV (C), normalized TV (D), and normalized RR (E). Baseline values were not different among treatment conditions for MV [F(10, 107) = 1.33; *P* = 0.22, two-way ANOVA], TV [F(10, 107) = 0.78; *P* = 0.65, two-way ANOVA], or RR [F(10, 107) = 0.86; *P* = 0.57, two-way ANOVA]. Solid lines represent time points that are significantly different than vehicle (*P* < 0.05; repeated measures two-way ANOVA with Dunnett’s multiple comparison test). (F) Maximum fold changes in RR and MV were determined for each mouse, averaged within each condition, and plotted as mean ± S.E.M. Details of statistical comparisons are provided in Supplemental Table 1.

To begin to explore the structure-function relationship of the aminopyridine scaffold, we used plethysmography to quantitatively screen the effects of commercially available derivatives on ventilatory function in mice at terminal stages of botulism. We selected nine derivatives of the 4-aminopyridine scaffold (Fig. 4A), containing substitutions at the 2, 3, and/or 3,5 positions, as well as deuterated 3,4-DAP and 4-aminopyridine (Fig. 4A). These analogs were chosen to help explore the effects of electron donating/withdrawing substituents on the ring nitrogen as well as the anticipated changes in the physicochemical properties. Because these studies were designed to quickly assess whether new aminopyridines have therapeutic effects in an established model of botulism, all analogs were evaluated at 2 mg/kg, which was found to be well tolerated in healthy mice for all experimental aminopyridines (Supplemental Table 2).

Most aminopyridines had robust effects on ventilatory parameters, including 3F-4AP, 2,4-DAP, 4-AP, 3,4,5-TAP, 4-AP-d4, 3,4-DAP-d3, and 2,4-DAP (Fig. 4, C–E). Although 3F-4AP significantly improved MV, symptomatic benefits were modest and short in duration compared with the other effective aminopyridines. On the contrary, 2F-4AP and 3,5DF-4AP were indistinguishable from vehicle, which we hypothesize is due to the altered negative logarithm of the acid dissociation constant (p*K*_a_) and consistent with data suggesting that the pyridine nitrogen must be protonated to block VGKC (Fig. 4B) (Kirsch and Narahashi, 1983; Howe and Ritchie, 1991; Muñoz-Caro and Niño, 2002; Caballero et al., 2007; Rodríguez-Rangel et al., 2020). Not surprisingly, the aminopyridines tested in this work exhibited apparent differences in the duration of the effect and the time to maximal response (Fig. 4, C–E), indicating possible differences in pharmacokinetics, cell permeability, and/or VGKC binding affinity. Although 2,4-DAP, 4-AP, 4-AP-d4, and 3,4,5-TAP increased TV and RR by roughly equal proportions, 3,4-DAP and 3,4-DAP-d3, which have low central nervous system (CNS) permeability (Lemeignan et al., 1984), had more potent effects on TV, whereas the CNS-permeable 3F-4AP had more potent effects on RR (Brugarolas et al., 2018) (Fig. 4F). The hysteresis between TV and RR suggests that some aminopyridines may have partially selective effects on central versus peripheral respiratory circuits. Although further studies are required to understand safety, toxicity, pharmacokinetic parameters, and dose-response effects for these aminopyridines, this functional screen suggests that the 4-aminopyridine scaffold is a flexible platform for the development of improved therapies for botulism.

To test whether aminopyridines could activate central respiratory circuits in healthy mice, mice were treated with 4-AP or 3,4-DAP and changes in TV, MV, and RR were monitored over 90 minutes by UWBP (Supplemental Fig. 1). 4-AP has approximately sixfold greater blood brain barrier permeability than 3,4-DAP (Lemeignan et al., 1984), and consequently would be expected to have more robust effects on ventilatory parameters if aminopyridines directly affected central respiratory circuits. Both analogs significantly increased RR, with concomitant reductions in TV and no changes in MV. The increased effects size elicited by 4-AP on RR compared to 3,4-DAP, when combined with the increased central access of 4-AP, suggests that aminopyridines can modulate central activity with variable efficiency depending, in part, on blood-brain barrier penetration. However, the relatively small effect of aminopyridines on RR in healthy mice compared to intoxicated mice suggests that direct activation of central respiratory circuits alone is not sufficient to reproduce therapeutic benefits.

## Discussion

Botulism symptoms are caused by reduced acetylcholine release from motor nerve terminals (Bradford et al., 2018). We have shown in rodent models that 3,4-DAP reverses the toxic signs of botulism and has antidotal effects when administered throughout the course of disease (Vazquez-Cintron et al., 2020; Machamer et al., 2022). Similarly, 3,4-DAP and 4-AP have shown clinical efficacy in anecdotal studies of patients with botulism (Ball et al., 1979; Dock et al., 2002; Friggeri et al., 2013). However, the specific effects of 3,4-DAP treatment on respiratory function in the context of botulism remain unknown. Here, we demonstrate that 3,4-DAP reverses respiratory acidosis by restoring ventilatory function at terminal stages of botulism in mice. Furthermore, we found that seven other 4-AP derivatives also reversed botulism symptoms, with varying efficacies. Some aminopyridines appear to have preferential effects on tidal volume versus respiratory rate, suggesting the differential engagement of multiple therapeutic mechanisms. These data demonstrate the therapeutic effects of 3,4-DAP and other aminopyridines on respiratory function in the context of botulism and illustrate the potential of aminopyridines as first-line treatments for respiratory weakness caused by central and peripheral diseases.

To our knowledge, this is the first study to evaluate the physiologic effects of botulism on ventilation and respiration. Our key finding is that 3,4-DAP treatment reverses BoNT/A-induced respiratory acidosis in a dose- and time-dependent manner. Prior to treatment, mice presented with an ∼80% reduction in minute volume and characteristic signs of respiratory acidosis, including increased *p*CO_2_, decreased blood pH, increased bicarbonate levels, and reduced lactate levels (Engel et al., 1967; Graham et al., 1986). Treatment of intoxicated mice with 3,4-DAP significantly improved ventilation and blood gases in a dose-dependent manner. Because 3,4-DAP Cmax is not affected by dose, the delayed improvement in blood gas levels at 0.25 mg/kg 3,4-DAP (45 minutes) versus 2 mg/kg 3,4-DAP (<30 minutes) is probably attributable to the slower accumulation of 3,4-DAP in motor neurons. Unexpectedly, we found that *p*O_2_ was not depressed in moribund mice exhibiting agonal breathing and a 90% reduction in minutes volume. The lack of change in *p*O_2_ suggests that hypercapnia and respiratory acidosis are the primary respiratory manifestations of botulism, with hypoxemia developing in the last minutes before death. This is particularly intriguing since the short- and long-term effects of hypercapnia-induced respiratory acidosis are not well understood, but are known to involve the cardiovascular, brain, metabolic, and respiratory systems (Almanza-Hurtado et al., 2022).

The direct effects of 3,4-DAP on tidal volume in vivo are consistent with mechanistic studies showing that 3,4-DAP reverses neuromuscular weakness in BoNT-intoxicated diaphragms by directly enhancing neuromuscular transmission (Bradford et al., 2018; Vazquez-Cintron et al., 2020). However, the data presented here and by Vazquez-Cintron et al. (2020) also found that aminopyridines with poor brain penetration, such as 2,4-DAP (Biessels et al., 1987; Damsma et al., 1988), 3,4,5-TAP (Mayorov et al., 2010), and 3,4-DAP (Lemeignan et al., 1984), still have robust effects on respiratory rate. This was unexpected because respiratory rate is regulated primarily by the CNS in response to chemoreceptors that measure *p*CO_2_ in the brain and blood (Tipton et al., 2017). One possible explanation for this apparent contradiction is that low concentrations of aminopyridines in the CNS can enhance respiratory signaling by potently inhibiting one or more VGKCs. In fact, 3,4-DAP and 4-AP are broad-spectrum VGKC blockers that can inhibit a wide variety of differentially localized VGKCs with various potencies (Judge and Bever, 2006), raising the possibility that different aminopyridines can have concentration-dependent effects on separate neurologic pathways. Indeed, previous studies have shown that 4-AP increases RR and reduces TV in conscious rabbits, in part through modulating central dopaminergic circuits (Böhmer et al., 1986). Alternatively, aminopyridines may stimulate peripheral networks that, in turn, modulate central respiratory circuits. For example, carotid chemoreceptors, which are the principal peripheral sensors of hypercapnia and acidosis (Iturriaga et al., 2021), express VGKCs that are highly sensitive to aminopyridine blockers (Zhou et al., 2016; Wang and Kim, 2018). It is intriguing to hypothesize that aminopyridines can activate carotid bodies to stimulate respiratory centers in the brain. Interestingly, carotid bodies express high levels of the BoNT/A molecular target (SNAP-25) and protein receptor (SV2) (Zhou et al., 2016) and thus, more speculatively, may be susceptible to BoNT intoxication. Blockade of carotid body signaling by BoNT would have important implications for disease progression, symptomatic manifestation, and recovery. Furthermore, carotid body intoxication may partially explain the therapeutic effects of aminopyridines on central respiratory drive. Consistent with this hypothesis is the finding that 4-AP and 3,4-DAP produce smaller increases in RR in healthy mice compared with intoxicated mice. A logical explanation for increased effects on RR in intoxicated mice is that aminopyridines restore neurotransmission at intoxicated synapses that regulate or influence central respiratory drive, such as the carotid bodies or reflex signaling from the diaphragm.

To address the need for aminopyridines with improved pharmaceutical properties, we used plethysmography to assess commercially available aminopyridines for therapeutic efficacy. In early studies with 3,4-DAP, we found UWBP was suitable to rapidly quantify therapeutic effects at clinically relevant stages of botulism in small cohorts with high fidelity and reproducibility. Based on this success, we used UWBP to evaluate structure-activity relationships among substituted derivatives of 4-AP. We found that substitutions at the 2, 3, and/or 3,5 positions can be tolerated without eliminating VGKC binding activity (Kirsch and Narahashi, 1978; Mayorov et al., 2010; Brugarolas et al., 2018; Rodríguez-Rangel et al., 2020). We took advantage of this steric flexibility to evaluate the in vivo effects of fluoro, amino, and methyl substitutions on ventilation in mice at terminal stages of botulism. These substitutions were expected to affect protonation (p*K*_a_) and lipophilicity (logP), which, in turn, will impact VGKC binding affinity and pharmacokinetic properties. Not surprisingly, most of the substituted aminopyridines retained their therapeutic effects, with pharmacodynamic durations that were similar to 3,4-DAP. Interestingly, aminopyridines exhibited a ∼threefold difference in tidal volume and a ∼twofold difference in respiratory rate, suggesting that UWBP is sufficiently sensitive to distinguish among mechanisms of action when evaluating small-molecule drugs. Although it is tempting to speculate on how individual substitutions affect tidal volume versus respiratory rate, elucidation of these differences requires a better understanding of the physicochemical properties of each aminopyridines, including VGKC selectivity and inhibition, pharmacokinetic profile, cell permeability, and CNS penetration. Nonetheless, the rapid and facile identification of aminopyridines utilizing UWBP that are active in vivo illustrates the power of our approach for drug discovery in botulism and other neuromuscular and respiratory diseases.

The therapeutic effects of aminopyridines are determined by several properties, including binding affinity to the VGKC, p*K*_a_ (which determines intracellular accumulation), lipophilicity at physiologic pH (logD, the partition coefficient between water and octanol), and pharmacokinetic parameters. In addition to testing 4-AP derivatives with known differences in lipophilicity and pK_a_, we also investigated whether deuteration of the pyridine ring could improve the pharmacodynamic effects of 3,4-DAP or 4-AP. Deuteration is a common approach to improve the pharmacology or stability of drugs (Hok et al., 2020; Di Martino et al., 2023). However, deuteration appears to have only a modest effect on the therapeutic effects of 3,4-DAP and 4-AP. In the case of 4-AP, although the primary metabolic product is 3-hydroxylation, likely via CYP2E1, the parent compound undergoes limited metabolic processing, with clearance primarily via urinary excretion of the parent compound (Blight and Henney, 2009; Caggiano and Blight, 2013). Accordingly, deuteration at the 3-position of 4-AP should have minimal effect on metabolism. In the case of 3,4-DAP, the primary route of metabolism is through acetylation of the 3-amino group, which would not be affected by deuteration. In this case, the question of whether the deutero-analog would modulate binding (Shi et al., 2018) to the VGKC was indirectly interrogated. Again, deuteration did not appear to significantly affect therapeutic efficacy, suggesting that any effects on binding were modest. These data suggest that deuteration neither enhances nor disrupts the therapeutic benefits of 4-AP and 3,4-DAP in treatment of botulism, but do not shed light on the effects of deuteration on other pharmacokinetic properties. 

This study has two main limitations. First, blood samples were taken from sedated mice using cardiac bleeds. Although cardiac samples were collected using a consistent methodology, the precise origin of the blood sample within the heart (e.g., venous versus arterial) is uncertain. Whereas venous versus arterial origin has negligible effects on *p*CO_2_, lactate, HCO_3_^−^, and pH values, *p*O_2_ is highly sensitive to the cardiac chamber (Razi et al., 2012; Byrne et al., 2014). To mitigate concerns about the effects of cardiac location and *p*O_2_ variability, we collected a relatively large number of samples to reduce the chances of a type II error. Nonetheless, it is possible that decreases in *p*O_2_ may be apparent at earlier stages of botulism and simply not reflected in our data. Second, although it is tempting to extrapolate pharmacokinetic properties from in vivo plethysmography data, botulism is a dynamic disease, and treatment effects are not measured under steady-state conditions. Interpretations of therapeutic effects are also influenced by molecular properties that vary among aminopyridines, such as electrical properties, binding affinities for different VGKCs, ion trapping, and lipophilicity. Consequently, without conducting a full characterization of pharmacokinetics and efficacies for each aminopyridine at multiple doses, it is inappropriate to extrapolate relative therapeutic values from single-dose studies. Finally, ventilation is a complex system modulated by dynamic set points that responds to changes in blood gas levels, such that an apparent reduction in minute volume may not represent a loss of treatment effects but rather sufficient recovery of blood gas levels to reduce respiratory drive. Accordingly, although in vivo data provide clear evidence of therapeutic potential, elucidating the underlying mechanisms requires additional studies.

In summary, we show that 3,4-DAP restores ventilation and reverses respiratory acidosis in mice after intravenous challenge with 2.2x LD_50_ BoNT/A, providing a therapeutic basis for the symptomatic and antidotal effects of 3,4-DAP observed in rodent botulism models (Vazquez-Cintron et al., 2020; Machamer et al., 2022). In addition to supporting the use of 3,4-DAP as a first-line treatment of botulism symptoms, these data are expected to contribute to the development of improved aminopyridines for the treatment of botulism or other indications involving neuromuscular weakness caused by impaired synaptic neurotransmission.

## Abbreviations

2,4-DAP: 2,4-diaminopyridine
3,4-DAP: 3,4-diaminopyridine
3,4-DAP-d3: deuterated 3,4-diaminopyridine
3F-4AP: 3-fluoro-4-aminopyridine
4-AP: 4-aminopyridine
BoNT: botulinum neurotoxin
CNS: central nervous system
LEMS: Lambert-Eaton myasthenic syndrome
LogP: logarithm of the partition coefficient of a solute between octanol and water
MV: minute volume
*p*CO_2_: arterial carbon dioxide partial pressure
pKa: negative logarithm of the acid dissociation constant
*p*O_2_: arterial oxygen partial pressure
RR: respiratory rate
SNARE: soluble *N*-ethylmaleimide–sensitive factor attachment protein receptor
3,4,5-TAP: 3,4,5-triaminopyridine
TV: tidal volume
UWBP: unrestrained whole-body plethysmography
VGKC: voltage-gated potassium channel
